# Velaglucerase alfa for Gaucher disease in Japan: Long-term post-marketing surveillance data from a unique population with high rates of neuronopathic subtypes

**DOI:** 10.1016/j.ymgmr.2026.101373

**Published:** 2026-10-29

**Authors:** Miyako Hoshino, Kei Murayama, Hiroyuki Ida, Erika Asano, Hiromichi Otaka

**Affiliations:** a Japan Medical Office, Takeda Pharmaceutical Company Limited, Tokyo, Japan; b Department of Pediatrics, Graduate School of Medicine, Juntendo University, Tokyo, Japan; c Diagnostics and Therapeutics of Intractable Diseases, Intractable Disease Research Center, Graduate School of Medicine, Juntendo University, Tokyo, Japan; d Department of Metabolism, Chiba Children's Hospital, Chiba City, Japan; e Trustee, The Jikei University School of Medicine, Tokyo, Japan; f Patient Safety & Pharmacovigilance Japan, Takeda Development Center Japan, Takeda Pharmaceutical Company Limited, Osaka, Japan

**Keywords:** Adverse drug reaction, Effectiveness, Enzyme replacement therapy, Gaucher disease, Neuronopathic, Velaglucerase alfa

## Abstract

Gaucher disease (GD) is a rare, autosomal recessive disease with reduced β-glucocerebrosidase activity, leading to symptoms including hepatosplenomegaly, anemia, thrombocytopenia, and bone disease. Velaglucerase alfa is a native human β-glucocerebrosidase preparation approved as enzyme replacement therapy for Type 1 GD in many countries and for all GD types, including neuronopathic Type 2 and Type 3, in Japan. In this report, we provide the final (up to 8 years) safety and somatic effectiveness results of an all-case, open-label, post-marketing surveillance study in Japan. Among all patients, the incidence of adverse drug reactions (ADRs) was 23.3% (14/60 patients) and serious ADRs was 8.3% (5/60 patients). In the safety analysis population (excluding patients who did not provide consent for publication), ADR incidence was 19.5% (8/41 patients), most commonly vomiting and positive antibody test (4.9% [2/41 patients] each); one patient experienced vomiting as a serious ADR. ADRs were more common in Type 2 (7/14 patients) compared with Type 1 (1/12 patients) and Type 3 (0/15 patients). Three patients (7.3%), all Type 2, had five infusion-related reactions of pyrexia, vomiting (serious), swelling of eyelid, and hypersensitivity; all resolved or recovered. In the effectiveness analysis population (patients with any effectiveness data; *n* = 39), hemoglobin concentration, platelet count, and plasma angiotensin-converting enzyme concentration improved by Week 48 in patients with no prior GD treatment and were maintained in patients with prior GD treatment. These results support long-term use of velaglucerase alfa in Japanese patients with all GD types, with safety and somatic effectiveness consistent with previous findings.

## 1 Introduction

Gaucher disease (GD) is a rare, autosomal recessive disease characterized by markedly reduced activity of the lysosomal enzyme β-glucocerebrosidase (GBA), resulting in hepatosplenomegaly, anemia, thrombocytopenia, and bone disease [1], [2], [3]. Three types of GD have been described based on clinical presentation. Type 1 is a non-neuronopathic form of GD and is the most common type found in Western countries [2], [4]. Type 2 and Type 3 are neuronopathic forms of GD with heterogeneous phenotypes associated with a broad range of neuronal impairments [1], [2], [3]. Enzyme replacement therapy (ERT) to supplement the residual GBA activity is generally recommended as treatment for patients with GD [3], [5], [6]. Velaglucerase alfa is an ERT with the same amino acid sequence as the naturally occurring GBA enzyme that is produced in a human cell line and is approved for Type 1 GD in the United States and Europe, and for the somatic manifestations of all GD types in Japan [7], [8], [9].

There are an estimated 211 GD cases in Japan [10]. Unlike in Western countries, neuronopathic Type 2 and Type 3 have been reported to account for 23.2% and 30.4%, respectively, of GD cases [10]. Moreover, Type 1 GD tends to be more severe in Japanese patients than in non-Japanese patients [11], [12]. These phenotypic differences are likely related to the relative prevalence of specific *GBA* mutations in different ethnic populations. The most common mutation among the Ashkenazi Jewish population, as well as non-Jewish European populations, is the N370S (p.Asn409Ser) mutation, which is associated with a milder Type 1 GD [4], [13]. In contrast, the L444P (p.Leu483Pro) mutation, which is linked to more severe forms of GD, is the most prevalent mutation in Japan [14], [15]. Despite these key differences, most reports of the safety and efficacy of ERT have focused on Type 1 GD, with limited evaluation in neuronopathic GD [5], [16], [17]. Given the unique characteristics of Japanese patients with GD, it is essential to examine the safety and effectiveness of ERT in this population.

Velaglucerase alfa was shown to be well tolerated and to maintain clinical stability over 2 years in Japanese patients with Type 1 or Type 3 GD who had switched from imiglucerase [18]. Given the limited number of patients in the Japanese clinical trial, an all-case post-marketing surveillance (PMS) was initiated after the drug's launch to evaluate the real-world safety and effectiveness of velaglucerase alfa in Japanese patients with GD. A previous interim analysis (maximum 6 years' follow-up) reported initial safety results with changes in platelet count as the only assessment of hematological effectiveness [19]. In this final report, we extend the follow-up to 8 years with a larger number of patients included. In addition to longer-term safety data, including by patient characteristics, we present more detailed somatic effectiveness data, including hemoglobin concentration, platelet count, and angiotensin-converting enzyme (ACE) concentration. These multifaceted assessments are needed to more comprehensively evaluate somatic effectiveness in this unique population. Overall, the results from the final analysis of this PMS provide a comprehensive overview of the long-term safety and somatic effectiveness of velaglucerase alfa in Japanese patients with all types of GD.

## 2 Materials and methods

### 2.1 Study design

This was an all-case, open-label, observational PMS study mandated by the Japanese regulatory authority and conducted in accordance with Good Post-marketing Surveillance Practice (GPSP) [20]. The primary objective of the PMS was to evaluate the long-term safety and somatic effectiveness of velaglucerase alfa in routine clinical practice. Protocol review by institutional review boards is not required for PMS studies in Japan. In addition, PMS studies in Japan are conducted as part of regulatory requirements and do not require individual informed consent according to the Japanese GPSP regulations. However, patients (or, in the case of children, their guardian/legal representative) were asked to provide informed consent for their data to be included in any publication. The study was registered at ClinicalTrials.gov (NCT03625882).

### 2.2 Study population

All patients with a confirmed diagnosis of GD (Type 1, 2, or 3) who were treated with velaglucerase alfa (Takeda Pharmaceutical Company Limited, Tokyo, Japan), regardless of age, sex, or previous treatment for GD, between September 2014 (launch of velaglucerase alfa) and December 2021 could be registered in the PMS. Patients were to receive velaglucerase alfa (60 U/kg) intravenously once every 2 weeks, according to the package insert.

### 2.3 Data collection

All baseline characteristics, safety data, and somatic effectiveness data were collected from medical records and recorded using paper case report forms (CRFs). Data were collected for up to 8 years after velaglucerase alfa was launched, i.e., until the end of September 2022. Final database lock was on November 1, 2023. Safety data included: adverse events (AEs), coded using the Medical Dictionary for Regulatory Activities, Japanese translation, Version 26.0; adverse drug reactions (ADRs), defined as AEs for which a causal relationship to velaglucerase alfa could not be ruled out, in the opinion of the physician; infusion-related reactions (IRRs) occurring within 24 h of velaglucerase alfa administration, which were of specific interest; and presence of anti-velaglucerase alfa antibodies. Antidrug antibody testing was performed at the physician's discretion. The presence of total anti-velaglucerase alfa antibodies (including immunoglobulin [Ig] G, IgE, IgA, and IgM) was assessed using a screening assay. Samples that tested positive for anti-velaglucerase alfa antibodies subsequently underwent antibody titer determination and further testing for neutralizing antibody activity. In addition, testing for IgE antibody activity could be performed upon physician request. The reasons for antibody testing were not collected. AEs, ADRs, and IRRs were categorized as serious if they: were life-threatening; resulted in death, persistent/significant disability, or congenital anomaly; required hospitalization or prolongation of hospitalization; or were otherwise medically important. An independent safety review was not conducted.

Somatic effectiveness data included hemoglobin concentration, platelet count, and plasma ACE concentration over time, and changes in spleen and liver volumes. All laboratory tests were performed by local laboratories at the treating physician's discretion (approximately every 12 weeks as a guide, although exact times could vary). Changes in spleen and liver volumes were also estimated at the treating physician's discretion (approximately every 24 weeks as a guide, but exact times could vary).

### 2.4 Statistical analysis

All data are reported descriptively with no inferential statistics. The safety analysis population included all patients who had CRFs collected and gave informed consent. For transparency, a limited amount of important safety data, such as serious ADRs, included patients who did not provide informed consent. Safety data are reported as number and percentage of patients for each type of event. ADRs are also reported for subgroups based on GD type and prior GD treatment. IRRs are described in detail for individual events. The effectiveness analysis population included all patients in the safety analysis population who had any recorded somatic effectiveness data. Effectiveness data are reported as mean (standard deviation [SD]) over time for the whole effectiveness analysis population and in subgroups based on the presence or absence of previous treatment for GD. All analyses were conducted using SAS 9.4 or later, or SAS Enterprise Guide (SAS Institute Japan) 8.2 or later.

## 3 Results

### 3.1 Demographic and baseline clinical characteristics

A total of 77 patients were enrolled at 58 medical sites; after excluding 14 patients whose enrollment was duplicated, the final number of enrolled patients was 63 (Fig. 1). CRFs were collected from 60 patients, and informed consent for publication was obtained from 41 patients who comprised the safety analysis population. The effectiveness analysis population included 39 patients with available effectiveness data.

**Fig. 1:**
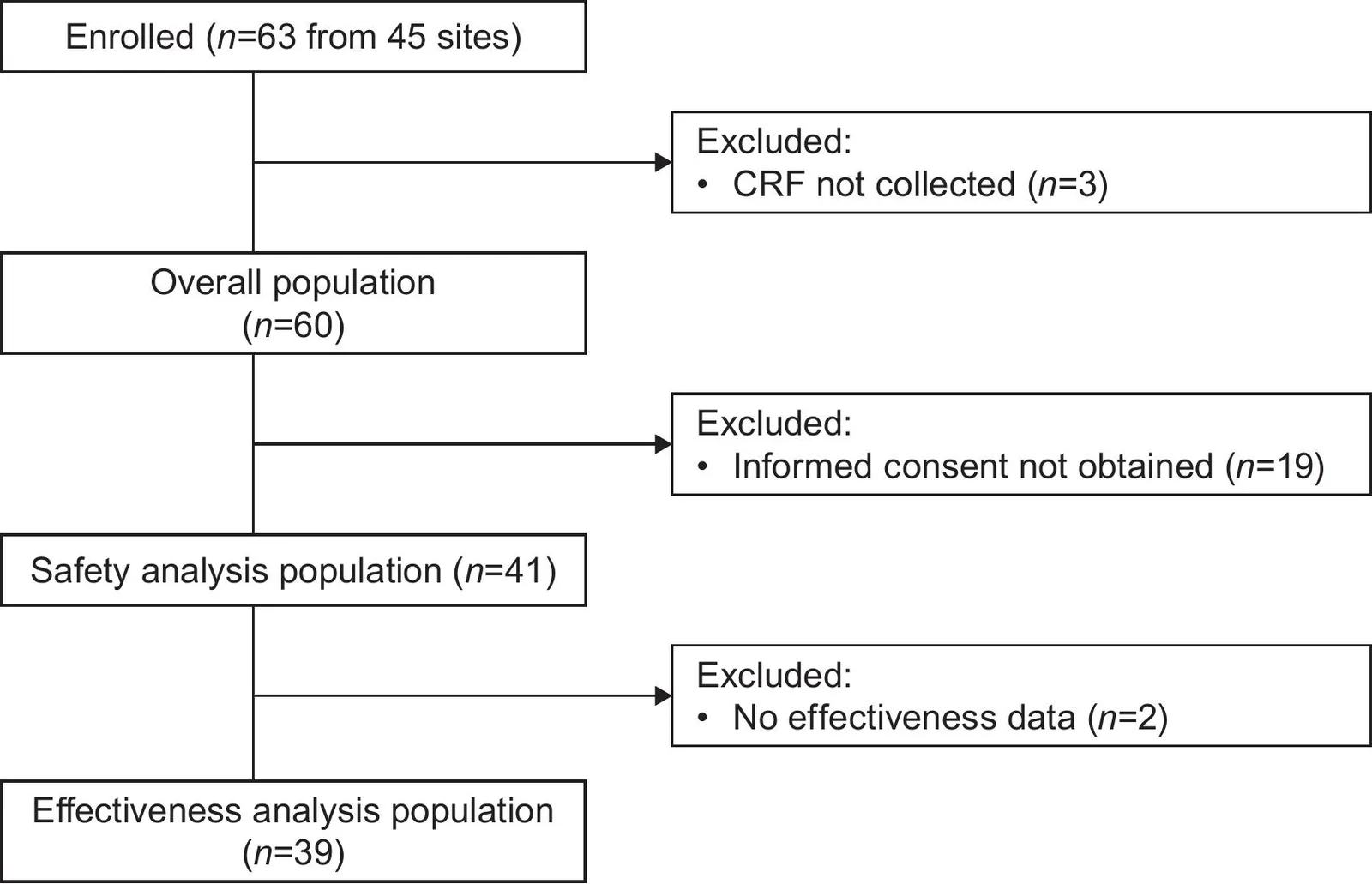
Patient disposition. CRF: case report form.

Within the safety analysis population (i.e., patients who provided informed consent), there were similar numbers of patients with each type of GD (Type 1, *n* = 12; Type 2, *n* = 14; Type 3, *n* = 15) (Table 1). The median (range) age at the start of velaglucerase alfa treatment was 49.5 (2.0–70.0) years for Type 1 GD (91.7% starting at age ≥ 18 years), 1.0 (0.0–13.0) years for Type 2 GD (64.3% starting at age < 4 years), and 17.0 (0.0–36.0) years for Type 3 GD (46.7% starting at age ≥ 18 years). Among patients with neuronopathic GD, the most common neurological symptoms present at diagnosis were myoclonus, abnormal eye movement, and increased muscle tone (10/14; 71.4% each) in patients with Type 2 GD, and myoclonus (8/15; 53.3%) and abnormal eye movement (5/15; 33.3%) in patients with Type 3 GD. The most frequent *GBA1* mutation was L444P (p.Leu483Pro) (14/41; 34.1%), followed by F213I (p.Phe252Ile) (4/41; 9.8%) and D409H (p.Asp448His) (3/41; 7.3%); more than half of patients (24/41; 58.5%) had other mutations identified. More than half (27/41; 65.9%) had received prior therapeutic agents for GD, mainly ERT. Demographics in the overall population (*N* = 60, including patients who did not provide informed consent) were generally similar to the demographics in the safety analysis population (data not shown).

**Table 1.** Patient demographics and background (safety analysis population).

Variable	Total (*N* = 41)	Type 1 GD (*N* = 12)	Type 2 GD (*N* = 14)	Type 3 GD (*N* = 15)
Sex				
Male	22 (53.7)	5 (41.7)	8 (57.1)	9 (60.0)
Female	19 (46.3)	7 (58.3)	6 (42.9)	6 (40.0)
Age at start of velaglucerase alfa treatment, years				
Mean (SD)	21.3 (21.3)	46.5 (20.1)	2.7 (3.6)	18.6 (8.6)
Median (min, max)	17.0 (0.0, 70.0)	49.5 (2.0, 70.0)	1.0 (0.0, 13.0)	17.0 (0.0, 36.0)
<4^a^	11 (26.8)	1 (8.3)	9 (64.3)	1 (6.7)
<18	23 (56.1)	1 (8.3)	14 (100.0)	8 (53.3)
18–64	16 (39.0)	9 (75.0)	0	7 (46.7)
≥65	2 (4.9)	2 (16.7)	0	0
Age at GD diagnosis, years				
*n*	37	11	13	13
Mean (SD)	13.1 (18.9)	32.7 (23.5)	0.2 (0.6)	9.5 (8.4)
Median (min, max)	2.0 (0.0, 69.0)	27.0 (2.0, 69.0)	0.0 (0.0, 2.0)	9.0 (0.0, 27.0)
Neurological symptoms at diagnosis^b^				
Myoclonus	18 (62.1)	–	10 (71.4)	8 (53.3)
Abnormal eye movement	15 (51.7)	–	10 (71.4)	5 (33.3)
Abnormal pupillary light reflex	5 (17.2)	–	5 (35.7)	0
Increased muscle tonus	11 (37.9)	–	10 (71.4)	1 (6.7)
Other	8 (27.6)	–	4 (28.6)	4 (26.7)
Mutation^b^				
L444P (p.Leu483Pro)	14 (34.1)	4 (33.3)	7 (50.0)	3 (20.0)
F213I (p.Phe252Ile)	4 (9.8)	2 (16.7)	1 (7.1)	1 (6.7)
D409H (p.Asp448His)	3 (7.3)	0	0	3 (20.0)
Other	24 (58.5)	3 (25.0)	10 (71.4)	11 (73.3)
Unknown	4 (9.8)	4 (33.3)	0	0
Family history of GD	8 (19.5)	1 (8.3)	1 (7.1)	6 (40.0)
Prior therapeutic agents				
Yes^b^	27 (65.9)	7 (58.3)	8 (57.1)	12 (80.0)
ERT	27 (65.9)	7 (58.3)	8 (57.1)	12 (80.0)
SRT	2 (4.9)	1 (8.3)	0	1 (6.7)
No	14 (34.1)	5 (41.7)	6 (42.9)	3 (20.0)

Data are *n* (%), unless otherwise noted.

ERT: enzyme replacement therapy; GD: Gaucher disease; max: maximum; min: minimum; SD: standard deviation; SRT: substrate reduction therapy.

a Data for patients <4 years of age were not obtained in clinical trials and are considered missing in the risk management plan for velaglucerase alfa.

b Multiple answers possible. Original wording in the case report form was “Methods of type 2 and type 3 GD diagnosis.”

In the safety analysis population, the mean (SD) initial dose of velaglucerase alfa was 58.9 (11.3) U/kg, the mean (SD) dose per administration was 59.8 (8.2) U/kg, and the mean (SD) final dose was 59.8 (8.4) U/kg, indicating that the dose was stable and was aligned with the package insert (Supplementary Table 1). Doses were similar in all three types of GD (Supplementary Table 1). At data cutoff, the mean (SD) duration of treatment with velaglucerase alfa in the safety analysis population was 56.1 (28.9) months (median 52.3 months), and the mean (SD) number of doses was 105.7 (56.3) (median 107.0 doses).

### 3.2 Safety

For full disclosure of important safety events, we report the incidence of ADRs and serious ADRs within the overall population (*N* = 60), including patients who did not provide informed consent for publication. The incidence of ADRs was 23.3% (14/60 patients), and the incidence of serious ADRs was 8.3% (5/60 patients), including vomiting, respiratory failure, peritonitis, ischemic colitis, eosinophilic gastroenteritis, and death. The ADR coded with the preferred term “death” and the ADR of respiratory failure each had a fatal outcome; these two ADRs were not included in the safety analysis population. All subsequent results are reported for the safety analysis population consisting of patients who consented to the use of their data in this publication.

In the safety analysis population (*N* = 41), the incidence of ADRs was 19.5% (8/41 patients) (Table 2). The most common ADRs were vomiting and positive antibody test (2/41 patients, 4.9% each). With respect to time of onset, no ADRs occurred within the first day of initiating velaglucerase alfa treatment. One patient each experienced vomiting and pyrexia within the first month of treatment, whereas thrombocytopenia, hypersensitivity, and hepatic function abnormality did not occur until after 1 year of treatment. Serious AEs occurred in 36.6% (15/41) of patients; the most common events were pneumonia, sepsis, urinary tract infection, myoclonic epilepsy, and vesicoureteral reflux in two patients (4.9%) each. Both patients who experienced myoclonic epilepsy had also had this symptom before initiating velaglucerase alfa treatment. As noted in the interim report [19], one patient (2.4%) discontinued velaglucerase alfa and the study because of a serious ADR of vomiting; no other serious ADRs occurred in the safety analysis population and no other patients discontinued due to an AE. The incidence of ADRs was higher in patients with Type 2 GD (7/14 patients; 50.0%) compared with Type 1 (1/12 patients; 8.3%) and Type 3 (0/15 patients; 0%) (Table 2). The incidence of ADRs was also higher in patients who had received prior therapeutic agents for GD (6/27 patients; 22.2%) than in those who did not (2/14 patients; 14.3%).

**Table 2.** Occurrence of ADRs by background category (safety analysis population).

		Prior therapeutic agents		GD type		
	Overall (*N* = 41)	Yes (*N* = 27)	No (*N* = 14)	Type 1 (*N* = 12)	Type 2 (*N* = 14)	Type 3 (*N* = 15)
Patients with ADRs, *n* (%)	8 (19.5)	6 (22.2)	2 (14.3)	1 (8.3)	7 (50.0)	0
Preferred Term						
Vomiting	2 (4.9)	2 (7.4)	0	0	2 (14.3)	0
Antibody test positive	2 (4.9)	2 (7.4)	0	0	2 (14.3)	0
Pyrexia	1 (2.4)	0	1 (7.1)	0	1 (7.1)	0
Thrombocytopenia	1 (2.4)	1 (3.7)	0	1 (8.3)	0	0
Hypersensitivity	1 (2.4)	1 (3.7)	0	0	1 (7.1)	0
Myoclonus	1 (2.4)	1 (3.7)	0	0	1 (7.1)	0
Swelling of eyelid	1 (2.4)	1 (3.7)	0	0	1 (7.1)	0
Hepatic function abnormal	1 (2.4)	1 (3.7)	0	0	1 (7.1)	0
Rash	1 (2.4)	1 (3.7)	0	0	1 (7.1)	0
AST increased	1 (2.4)	1 (3.7)	0	0	1 (7.1)	0
Hyperchlorhydria	1 (2.4)	0	1 (7.1)	0	1 (7.1)	0

Data are *n* (%), unless otherwise noted.

ADR: adverse drug reaction; AST: aspartate aminotransferase; GD: Gaucher disease.

Seven patients in the safety analysis population had AEs leading to death. These fatal AEs were assessed by the treating physicians as not being related to velaglucerase alfa. The reported deaths were one patient with Type 1 GD (acute myeloid leukemia and myelodysplastic syndrome), three patients with Type 2 GD (myoclonic epilepsy and asphyxia; central apnea syndrome; acute renal failure), and three patients with Type 3 GD (head injury caused by traffic accident; congestive heart failure; aspiration pneumonia and deterioration of general health condition).

Three patients (7.3%), all with Type 2 GD, experienced five IRRs of pyrexia (two events), vomiting, swelling of eyelid, and hypersensitivity (one event each) (Table 3). Two patients had two IRRs each, and two patients had received previous treatment for GD. One IRR of vomiting was serious and required hospitalization; this patient discontinued velaglucerase alfa. One patient (Patient 2) who had swelling of the eyelid and hypersensitivity as IRRs tested positive for anti-velaglucerase alfa antibodies. The time from first administration of velaglucerase alfa to IRR onset ranged from 2 days to more than a year. The outcome of all IRRs was recovered/resolved.

**Table 3.** IRRs in individual patients.

Patient	GD type	Prior treatment for GD	IRR Preferred Term	IRR onset timing^a^	Serious	Severity	Outcome
Patient 1	Type 2	Yes (ERT)	Vomiting	99 days	Yes (hospitalization)	Grade 2 (moderately severe)	Recovered/resolved
Patient 2	Type 2	Yes (ERT)	Swelling of eyelid	55 days	No	Grade 1 (mild)	Recovered/resolved
			Hypersensitivity	435 days	No	Grade 2 (moderately severe)	Recovered/resolved
Patient 3	Type 2	No	Pyrexia	2 days	No	Grade 1 (mild)	Recovered/resolved
			Pyrexia	16 days	No	Grade 1 (mild)	Recovered/resolved

ERT: enzyme replacement therapy; GD: Gaucher disease; IRR: infusion-related reaction.

a Time to IRR onset from first velaglucerase alfa administration.

Anti-velaglucerase alfa antibodies were detected in two of 13 patients tested. Of these two patients, one underwent additional testing for neutralizing antibodies and the result was negative, while the other underwent additional testing for IgE antibody activity and the result was positive. Both patients had received continued treatment for > 6 years by the end of the observation period, with no notable impact on somatic effectiveness due to antibody formation (data not shown). Several AEs (anemia, decreased white blood cell count, bronchitis, hypersensitivity, liver disorder, respiratory tract infection, and zinc deficiency) were reported following positive antibody tests in these two patients. Among these events, the only ADR was hypersensitivity, which was also classified as an IRR (Patient 2 in Table 3). However, the causal relationship between antibody positivity and the occurrence of these AEs is unknown.

### 3.3 Somatic effectiveness

In the effectiveness analysis population, among patients with no prior GD treatment, mean hemoglobin concentration increased from ∼10 g/dL at baseline to ∼13 g/dL at Week 48 and remained stable thereafter (Fig. 2). Similarly, mean platelet count increased from ∼100 × 10^9^/L at baseline to ∼220 × 10^9^/L at Week 24 and then remained stable at ∼200–300 × 10^9^/L through Week 384 (Fig. 3). Mean plasma ACE concentration decreased from ∼60 IU/L at baseline to ∼30 IU/L at Week 36 and slowly decreased further thereafter (Fig. 4). Among patients with prior GD treatment, hemoglobin, platelets, and ACE were generally maintained at baseline levels over the long term (Fig. 2, Fig. 3, Fig. 4). The number of patients with spleen and liver volume measurements was small (≤ 6 patients), and no clear trend in changes over time was observed; however, organ volumes were generally maintained (data not shown).

**Fig. 2:**
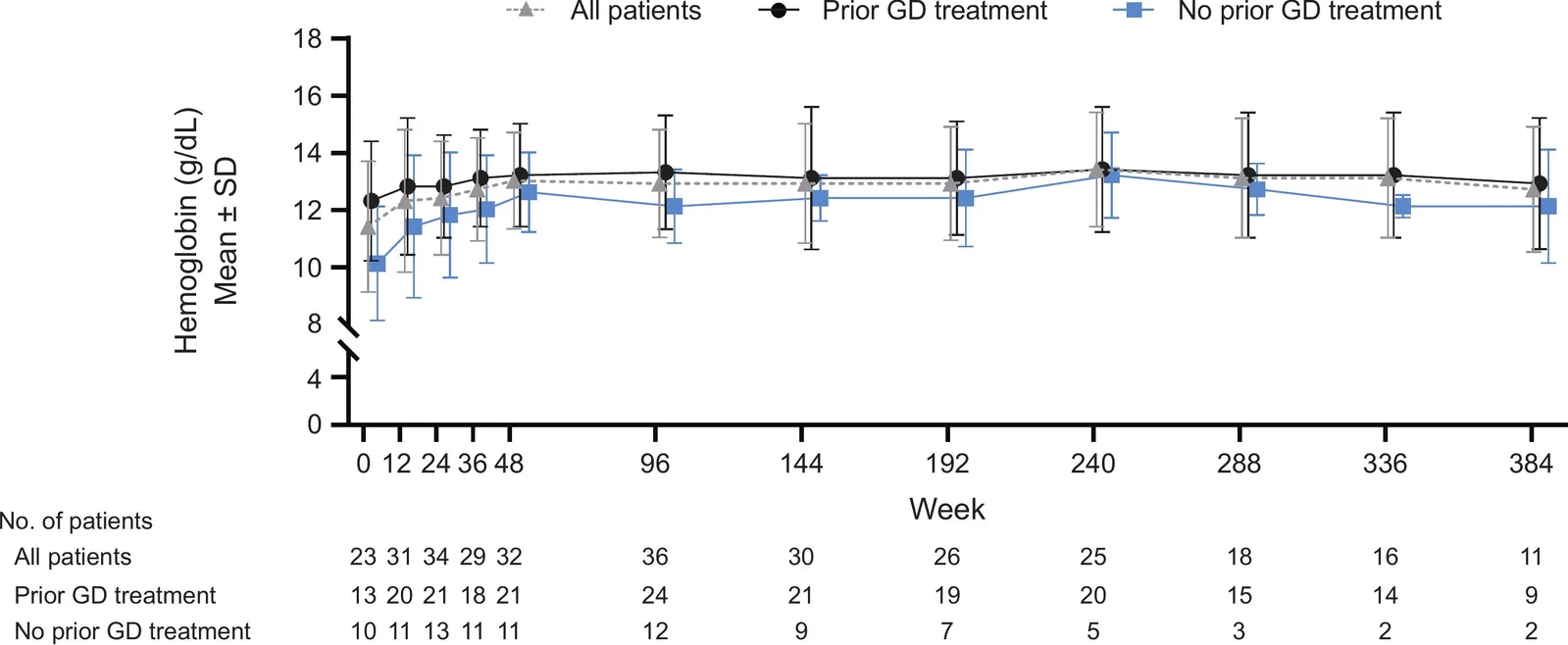
Hemoglobin concentration over time in the effectiveness analysis population. GD: Gaucher disease; SD: standard deviation.

**Fig. 3:**
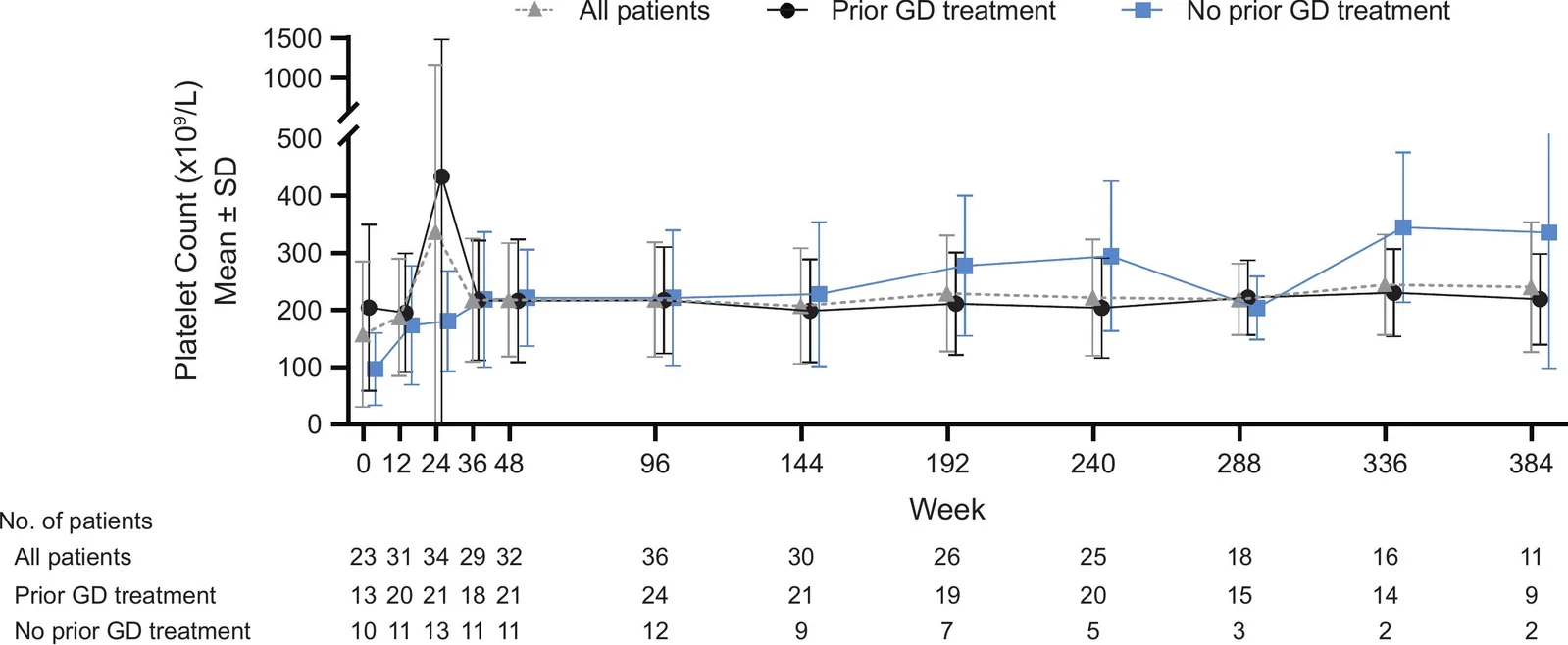
Platelet count over time in the effectiveness analysis population. GD: Gaucher disease; SD: standard deviation.

**Fig. 4:**
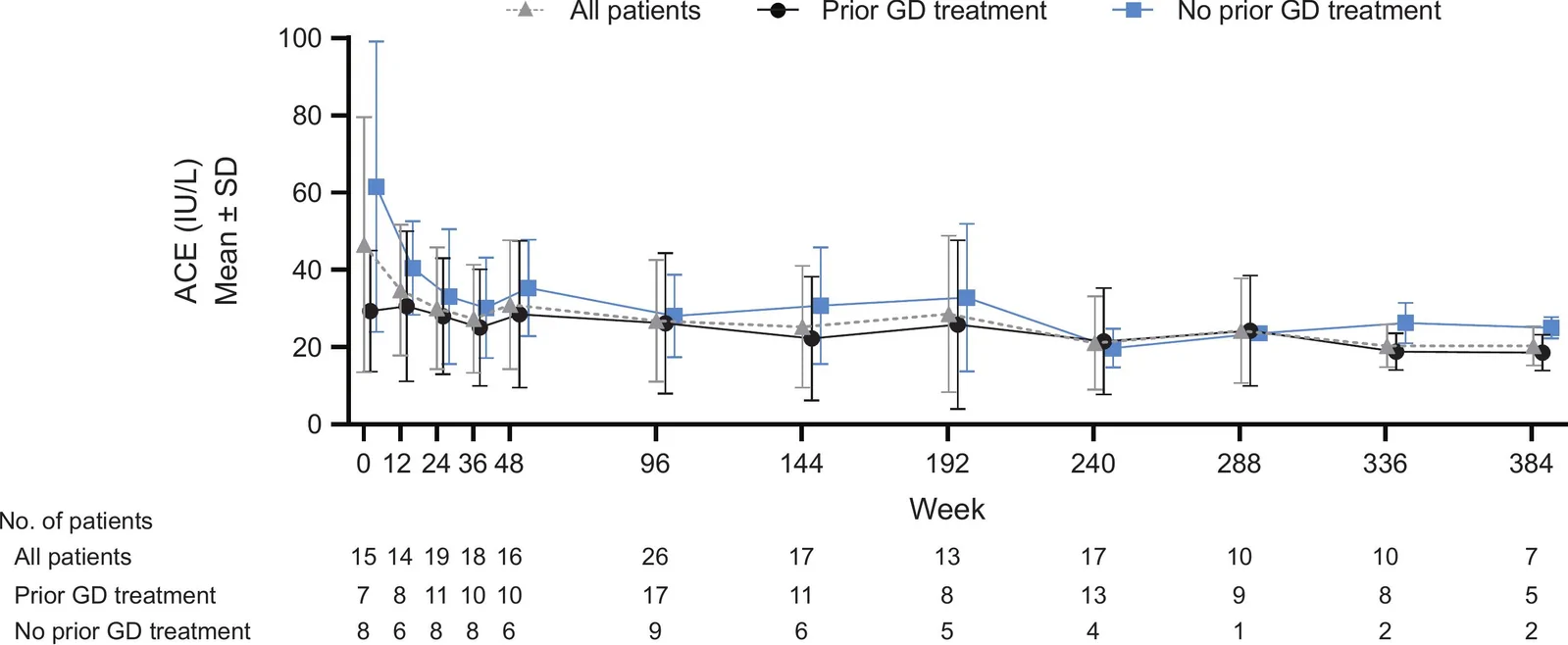
Plasma ACE concentration over time in the effectiveness analysis population. ACE: angiotensin-converting enzyme; GD: Gaucher disease; SD: standard deviation.

## 4 Discussion

This PMS study evaluated the long-term safety and somatic effectiveness of velaglucerase alfa in Japanese patients with GD. The PMS involved the longest follow-up time (up to 8 years) and the largest sample of patients of any study conducted in Japan. The study included patients with all three types of GD and across a broad range of ages. The results are consistent with the interim analysis of the study [19] and support the continued safety and somatic effectiveness of velaglucerase alfa in Japanese patients with all types of GD, including patients with neuronopathic GD, over the long term.

No new safety concerns were identified with up to 8 years of velaglucerase alfa treatment. The incidence of ADRs did not increase between the 6-year interim report (23.3%; 7/30 patients) [19] and this 8-year final report (23.3% [14/60 patients] in the overall population and 19.5% [8/41 patients] in the safety analysis population of patients who provided informed consent). This incidence was generally lower than the incidence of drug-related AEs observed in Phase 3 trials conducted overseas [21], [22], [23], [24], [25] and in Japan [18], [26]; however, comparisons with clinical trials are inherently limited due to differences in study settings such as the intensity of safety monitoring and physician-dependent AE reporting. In overseas trials conducted exclusively in patients with Type 1 GD, the incidence of drug-related AEs over 9–12 months ranged from 47% to 60% in treatment-naïve patients and from 14% to 36% in patients who had switched from imiglucerase [21], [23], [25]. In the long-term (up to approximately 5 years) extensions of these trials, drug-related AE incidence decreased to between 21% and 28% [22], [24], similar to the incidence of ADRs observed in this PMS. In the Japanese trial, which included patients with Type 1 or Type 3 GD, drug-related AEs occurred in three of five patients across both the parent trial and the extension over a total 24-month study period [18], [26]. In this PMS, patients with Type 2 GD had the highest incidence of ADRs (50.0%; 7/14 patients) compared with Type 1 GD (8.3%; 1/12 patients) and Type 3 GD (0%; 0/15 patients). As noted, Type 2 is the most severe type of GD, but some of the ADRs observed may be related to the severity of the underlying disease. Indeed, some patients with Type 2 GD already exhibited disease-related manifestations at the start of velaglucerase alfa treatment, including myoclonus, abnormal eye movements, and increased muscle tone. Although Type 2 GD has traditionally been considered to be fatal by around 1–3 years of age [2], some evidence suggests that ERT may prolong life in these patients [27]. Therefore, the findings of the present study highlight the importance of careful monitoring for ADRs during long-term treatment of patients with Type 2 GD who achieve prolonged survival. The low incidence of ADRs in patients with Type 1 GD is consistent with results from the Gaucher Outcome Survey (GOS), a real-world clinical practice registry currently involving more than 2000 patients (>90% Type 1 GD) in non-Japanese countries [28], [29]. In 376 patients who had only ever received velaglucerase alfa, drug-related AEs occurred in 8.8% of patients after a mean of 6.6 years [28]. In the 12-year GOS analysis, drug-related AEs occurred in 3.3% of 886 patients who had received velaglucerase alfa (with or without prior ERT) [29]. However, given the differences in patient population and study design, comparisons of incidence rates in this study and the GOS should be interpreted with caution.

IRR and antibody formation are important considerations with ERT, and no new findings were observed in this PMS. Three patients (7.3%) had a total of five IRRs; this incidence is lower than that seen in the global Phase 3 studies and extensions (range 10.5–56%) [21], [22], [23], [24], [25]. Vomiting was the only serious IRR, leading to hospitalization and discontinuation of velaglucerase alfa. Most (4/5) IRRs occurred within the first 4 months of treatment, and all five IRRs resolved or recovered. The incidence of IRRs was lower than that seen in the interim analysis (10.0%; 3/30 patients) [19] and slightly higher than that seen in the GOS cohort (5.9%; 22/376 patients), which consisted of > 90% patients with Type 1 GD and no patients with Type 2 GD [28]. Two patients developed anti-velaglucerase alfa antibodies, which did not appear to affect the safety or somatic effectiveness of the treatment, and both patients continued receiving velaglucerase alfa through the end of the observation period. However, because antibody testing was not mandatory, the actual incidence of antidrug antibodies is not clear.

The results of the hematological parameters indicate the long-term somatic effectiveness of velaglucerase alfa in this unique Japanese population. Hemoglobin concentration, platelet count, and plasma ACE concentration improved by Week 48 in patients with no prior GD treatment, whereas these parameters were maintained in patients with prior GD treatment. Although spleen and liver volumes were recorded for only a few patients, organ volumes appeared to be maintained. Previous studies have reported that ERT improves non-neuronopathic manifestations, including low hemoglobin, low platelet count, and enlarged organ volumes, in patients with Type 3 GD [28], [30], [31], [32], [33], which is consistent with our findings. Notably, 34.1% (14/41 patients) of the population in this PMS were patients with the severe Type 2 GD—a subgroup for which the somatic effectiveness of ERT has rarely been explored. Taken together, the results of this PMS indicate that velaglucerase alfa is of clinical value to Japanese patients with neuronopathic GD, including a high proportion of Type 2 GD, by improving the hematological symptoms, and possibly organomegaly.

This study confirmed that dosing of velaglucerase alfa in Japanese patients with GD is consistent with the recommended dose in the package insert. Previous reports from the GOS suggest that doses tend to be lower in Western countries, with marked differences between countries [28], [29], [34]. For example, most patients in Israel received <20 U/kg, most patients in the United Kingdom received 20–40 U/kg, and most patients in the United States received 60 U/kg [34]. These lower doses may reflect the higher prevalence of the milder Type 1 GD, especially in Israel, compared with Japan [28], [29], [34]. Therefore, because the Japanese patient population includes a greater proportion of severe cases resulting from the higher proportion of neuronopathic subtypes and higher rate of the L444P (p. Leu483Pro) mutation, there is a strong clinical motivation to maintain high dosing over the long term in real-world practice.

This is the largest study of velaglucerase alfa conducted in Japan, with an increase in sample size from 30 to 41 since the interim analysis [19]. This sample represents approximately 20% of the estimated total number of Japanese patients with GD [10]. The PMS included patients of various ages and similar numbers of patients with Types 1, 2, and 3 GD, reflecting the relative prevalence of these phenotypes in Japan. This final analysis used an 8-year observation period to assess long-term safety and somatic effectiveness, increased from 6 years at the interim analysis. The final analysis measured several somatic effectiveness measures, including hemoglobin concentration, platelet count, and ACE concentration, whereas the interim analysis only assessed platelet count. In addition, these somatic effectiveness outcomes were evaluated in subgroups of patients with and without prior GD treatment, which was not analyzed in the interim report. Furthermore, this analysis reported crucial safety data that included patients who did not provide informed consent for publication, which supports a balanced assessment of the benefit-risk ratio of velaglucerase alfa. Taken together, this final analysis provides a more comprehensive evaluation than the 6-year interim analysis.

Despite this study's strengths, several limitations must be acknowledged. First, the sample size was limited, especially for subgroups based on GD type and prior therapy. However, sample size will always be limited by the rarity of GD. Because patients were treated and assessed as part of regular clinical practice, somatic effectiveness data were missing for some patients at some time points, and bone mineral density and organ volume data were only available in a few patients, while other outcomes of interest, such as neurological assessments, growth and development in children, and quality of life, could not be evaluated in the context of this PMS. In addition, although ACE is relatively non-specific for GD activity compared with newer biomarkers such as glucosylsphingosine (lyso-Gb1), chitotriosidase, and CCL18 [35], it is more commonly used in Japan because the cost is reimbursed by insurance, whereas the cost of newer biomarkers is not. Because the causal relationship between velaglucerase alfa treatment and adverse events was determined by the treating physician, it is possible that some manifestations of worsening disease were reported as ADRs, particularly for Type 2 GD, the most severe and rapidly progressing form. The study did not include a comparator group of patients treated with other therapies or untreated patients. Finally, most outcomes were analyzed only in the populations that provided informed consent for publication and therefore may not be fully representative. To overcome these limitations, robust data from a study conducted in a more controlled setting are needed, especially in patients with neuronopathic GD.

## 5 Conclusion

The results of the 8-year final analysis of this PMS were consistent with the results of the interim analysis. No new safety concerns were identified. Somatic effectiveness, assessed by hemoglobin, platelet count, and ACE, improved in patients with no prior GD treatment, whereas these parameters were maintained in patients with prior GD treatment. These results support the long-term use of velaglucerase alfa in a unique population of Japanese patients with prevalent neuronopathic GD, with safety and somatic effectiveness consistent with previous findings.

## CRediT authorship contribution statement

**Miyako Hoshino:** Writing – review & editing, Writing – original draft, Project administration, Formal analysis. **Kei Murayama:** Writing – review & editing, Investigation. **Hiroyuki Ida:** Writing – review & editing, Investigation. **Erika Asano:** Writing – review & editing, Validation. **Hiromichi Otaka:** Writing – review & editing, Writing – original draft, Methodology.

## Funding

This study was funded by Takeda Pharmaceutical Company Limited. Takeda was involved in the study design, data collection, and data analysis, and Takeda authors were involved in the development of the manuscript content. All authors had access to the data. Medical writing assistance was provided by Rebecca Lew, PhD, CMPP, and Hana Nomura, BPharm (Hons), CMPP, of ProScribe KK, part of Envision Ignite, an Envision Medical Communications agency, a part of Envision Pharma Group, and was funded by Takeda. ProScribe's services complied with international guidelines for Good Publication Practice.

## Declaration of competing interest

The authors declare the following financial interests/personal relationships which may be considered as potential competing interests: Miyako Hoshino reports financial support, administrative support, article publishing charges, and writing assistance were provided by Takeda Pharmaceutical Company Limited. Kei Murayama reports financial support, administrative support, article publishing charges, and writing assistance were provided by Takeda Pharmaceutical Company Limited. Hiroyuki Ida reports financial support, administrative support, article publishing charges, and writing assistance were provided by Takeda Pharmaceutical Company Limited. Erika Asano reports financial support, administrative support, article publishing charges, and writing assistance were provided by Takeda Pharmaceutical Company Limited. Hiromichi Otaka reports financial support, administrative support, article publishing charges, and writing assistance were provided by Takeda Pharmaceutical Company Limited. Miyako Hoshino reports a relationship with Takeda Pharmaceutical Company Limited that includes: employment. Erika Asano reports a relationship with Takeda Pharmaceutical Company Limited that includes: employment. Hiromichi Otaka reports a relationship with Takeda Pharmaceutical Company Limited that includes: employment. Kei Murayama reports a relationship with Takeda Pharmaceutical Company Limited that includes: speaking and lecture fees. Hiroyuki Ida reports a relationship with Takeda Pharmaceutical Company Limited that includes: honoraria and travel expenses for serving as a chairperson. Kei Murayama reports a relationship with Sanofi KK that includes: speaking and lecture fees. Hiroyuki Ida reports a relationship with Sanofi KK that includes: consultancy agreement, as well as honoraria and travel expenses for attending meetings. Hiroyuki Ida reports a relationship with JCR that includes: honoraria and travel expenses for serving as a chairperson. If there are other authors, they declare that they have no known competing financial interests or personal relationships that could have appeared to influence the work reported in this paper.

## Data Availability

The datasets generated during and/or analyzed during the current study are available from the corresponding author on reasonable request.

## Appendix A Supplementary data

Details of velaglucerase alfa dosing (safety analysis population).
