# Supplementary material for: Velaglucerase alfa for Gaucher disease in Japan: Long-term post-marketing surveillance data from a unique population with high rates of neuronopathic subtypes
Source: Mol Genet Metab Rep. 2026 Oct 29;49:101373. doi: 10.1016/j.ymgmr.2026.101373 (PMC13738124; doi:10.1016/j.ymgmr.2026.101373)
Supplement: Supplementary file 1 [file mmc1.pdf]

Supplementary data

Velaglucerase alfa for Gaucher disease in Japan: Long-term post-marketing surveillance data from a unique population with high rates of neuronopathic subtypes

Miyako Hoshino[a,*], Kei Murayama[b, c, d], Hiroyuki Ida[e], Erika Asano[f], Hiromichi Otaka[a]

AUTHORS' AFFILIATIONS

[a] *Japan Medical Office, Takeda Pharmaceutical Company Limited, Tokyo, Japan*

[b] *Department of Pediatrics, Graduate School of Medicine, Juntendo University, Tokyo, Japan*

[c] *Diagnostics and Therapeutics of Intractable Diseases, Intractable Disease Research Center, Graduate School of Medicine, Juntendo University, Tokyo, Japan*

[d] *Department of Metabolism, Chiba Children's Hospital, Chiba City, Japan*

[e] *Department of Pediatrics, The Jikei University School of Medicine, Tokyo, Japan*

[f] *Patient Safety & Pharmacovigilance Japan, Takeda Development Center Japan, Takeda Pharmaceutical Company Limited, Tokyo, Japan*

***CORRESPONDING AUTHOR**

Miyako Hoshino
Japan Medical Office
Takeda Pharmaceutical Company Limited
1-1, Nihonbashi-Honcho 2-Chome, Chuo-Ku
Tokyo, Japan 103-8688
Email: miyako.hoshino@takeda.com

CONTENTS

Supplementary Table 1

Details of velaglucerase alfa dosing (safety analysis population).

Supplementary Table 1

Details of velaglucerase alfa dosing (safety analysis population).

Variable	Total (*N*=41)	Type 1 GD (*N*=12)	Type 2 GD (*N*=14)	Type 3 GD (*N*=15)
Initial dose, U/kg				
n	40	11	14	15
Mean (SD)	58.9 (11.3)	55.5 (8.9)	62.8 (12.6)	57.8 (11.3)
Median (min, max)	60.0 (19.2, 93.8)	59.3 (37.3, 65.9)	60.5 (50.0, 93.8)	60.0 (19.2, 67.6)
Average dose per administration, U/kg				
n	41	12	14	15
Mean (SD)	59.8 (8.2)	56.9 (6.3)	62.7 (11.6)	59.4 (4.5)
Median (min, max)	59.7 (35.9, 81.4)	58.7 (37.3, 61.1)	60.7 (35.9, 81.4)	59.7 (51.8, 67.6)
Final dose, U/kg				
n	32	12	11	9
Mean (SD)	59.8 (8.4)	58.0 (9.1)	60.6 (8.1)	61.0 (8.5)
Median (min, max)	59.7 (37.3, 77.9)	56.9 (37.3, 77.9)	61.5 (43.5, 69.9)	60.4 (50.9, 76.2)

GD: Gaucher disease; max: maximum; min: minimum; SD: standard deviation.
